# Biochar from Co-Pyrolyzed Municipal Sewage Sludge (MSS): Part 1: Evaluating Types of Co-Substrates and Co-Pyrolysis Conditions

**DOI:** 10.3390/ma17143603

**Published:** 2024-07-21

**Authors:** Michael Biney, Mariusz Z. Gusiatin

**Affiliations:** Department of Environmental Biotechnology, Faculty of Geoengineering, University of Warmia and Mazury in Olsztyn, Sloneczna Str. 45G, 10-709 Olsztyn, Poland; michael.biney@uwm.edu.pl

**Keywords:** municipal sewage sludge, co-pyrolysis, biomass, waste, biochar

## Abstract

With the increasing production of municipal sewage sludge (MSS) worldwide, the development of efficient and sustainable strategies for its management is crucial. Pyrolysis of MSS offers several benefits, including volume reduction, pathogen elimination, and energy recovery through the production of biochar, syngas, and bio-oil. However, the process can be limited by the composition of the MSS, which can affect the quality of the biochar. Co-pyrolysis has emerged as a promising solution for the sustainable management of MSS, reducing the toxicity of biochar and improving its physical and chemical properties to expand its potential applications. This review discusses the status of MSS as a feedstock for biochar production. It describes the types and properties of various co-substrates grouped according to European biochar certification requirements, including those from forestry and wood processing, agriculture, food processing residues, recycling, anaerobic digestion, and other sources. In addition, the review addresses the optimization of co-pyrolysis conditions, including the type of furnace, mixing ratio of MSS and co-substrate, co-pyrolysis temperature, residence time, heating rate, type of inert gas, and flow rate. This overview shows the potential of different biomass types for the upgrading of MSS biochar and provides a basis for research into new co-substrates. This approach not only mitigates the environmental impact of MSS but also contributes to the wider goal of achieving a circular economy in MSS management.

## 1. Introduction

**Figure 1:**
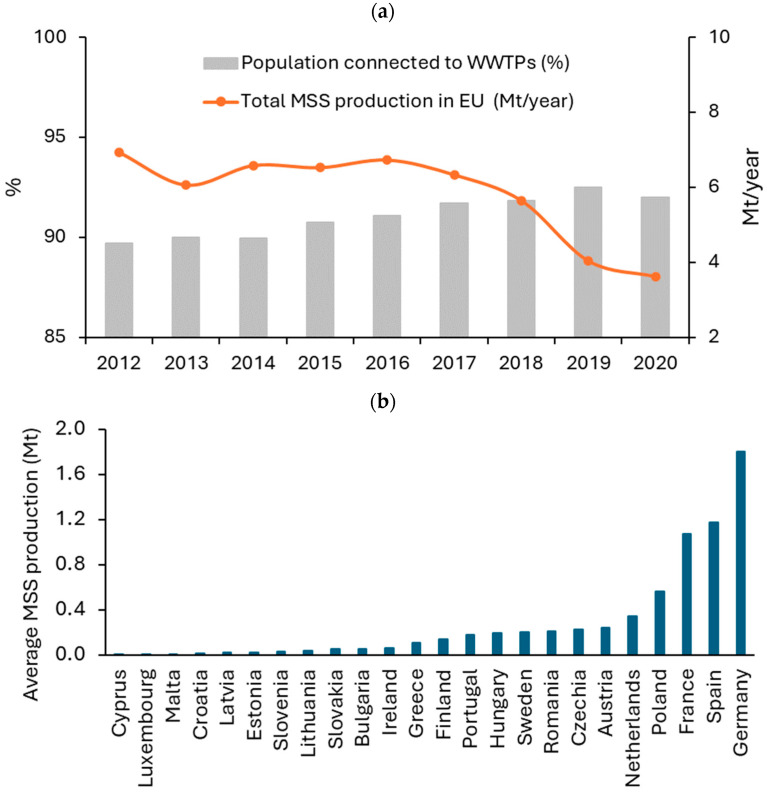
The MSS production in the EU countries: (**a**) total MSS production with percentage of population connected to WWTPs, (**b**) MSS production in EU countries (average from 2012 to 2020) [10].

Global MSS production is a serious concern requiring prompt and decisive action. The rapid growth in population, urbanization, and industrialization has led to a significant increase in the volume of wastewater generated, resulting in an increase in MSS production. In 2017, the global sewage sludge production was 45 Mt/year and is still increasing [1]. As of 2020, the approximate amount of municipal wastewater produced was estimated to be between 360 and 380 km^3^ [2], and according to the latest projections, it is likely to increase by 24% by 2030 and 51% by 2050 [2,3]. Europe, East Asia, and North America are the major contributors to global sewage sludge production [4]. Looking forward, it is crucial to consider the impact of sewage sludge production in the Southeastern Asian Nations, which could reach 20–24 Mt/year by 2050 [5]. The annual production of MSS in China has increased by a total of 10.7% since 2008. In 2019, the production of MSS in this country reached 11.75 Mt, and in 2020, the production of activated sludge had already exceeded 36 Mt [6,7,8]. The MSS production in Bangkok, Thailand, is noteworthy, with daily outputs ranging from 30,000 to 350,000 m^3^/d in multiple treatment plants across the city [9]. The MSS is a byproduct that is generated during the treatment of wastewater in municipal wastewater treatment plants (WWTPs). The composition and quality of the MSS depend on the extent to which the local population is connected to the treatment plant. Typically, at least secondary treatment is required. According to the Eurostat database, there is an increasing trend in the percentage of the population connected to WWTPs, and nowadays, it is over 90% (Figure 1a). In the European Union (EU), at least 95% of the population is served by WWTPs in six countries (Austria, Spain, the Netherlands, Luxembourg, Malta, and Germany). In contrast, the lowest percentages are served by WWTPs in Croatia (37%) and Romania (48%).

Annual MSS production in EU countries has shown some fluctuations in recent years, from 6.9 Mt in 2012 to 5.6 Mt in 2018 (Figure 1a). A certain downward trend in MSS production in recent years is due more to the amount of data reported by individual countries and does not reflect actual production. Based on figures for annual MSS production, Poland, France, Spain, and Germany contribute the most to MSS production among EU countries (Figure 1b).

**Figure 2:**
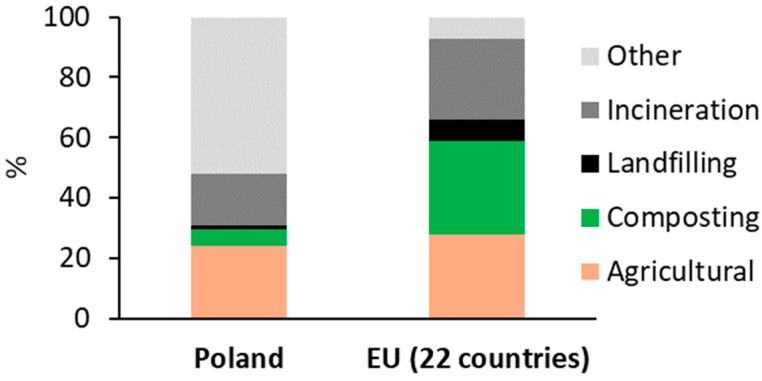
Comparison of the application of main methods for MSS management in Poland and the EU in 2022 [12].

The management of MSS involves challenges due to its large volume, high moisture content, complex composition, and the presence of contaminants. However, despite these issues, MSS displays promise as a valuable resource owing to its substantial content of organic matter and nutrients [11]. Various MSS treatment technologies have evolved over the years, differing across regions of the world. Currently, MSS in Europe is dealt with mainly by agricultural application, development of plants for compost production (composting), thermal conversion (mainly incineration), landfilling, and other methods, such as storage of MSS in WWTPs, stabilization, anaerobic digestion, etc. It is worth mentioning that in some EU countries, such as Belgium, the use of MSS for agricultural purposes is restricted by legal measures. This restriction is due to the existing nitrogen surplus in soils and waters. A comparison of the methods for MSS management in Poland and in the EU countries is shown in Figure 2 [12], while the frequency of application of MSS treatment methods in different countries is compared in Table 1.

Thermochemical treatments such as torrefaction, pyrolysis, and gasification are new methods of effectively reducing MSS volume, generating several products in a short time, and offering opportunities for energy recovery [11]. Recently, interest in the pyrolysis of MSS as a feedstock for bioenergy production has increased. Although pyrolysis is an acceptable waste disposal method due to its product selectivity, process scalability, and versatility, its effectiveness with MSS is highly dependent on MSS composition. This can vary based on the loading capacity of the WWTP, seasonal fluctuations in wastewater, and the treatment method [15]. One of the important products of pyrolysis is biochar. Overall, biochar derived from various feedstocks offers numerous benefits and has diverse applications, such as CO_2_ capture and storage, energy production, soil enhancement, adsorption of pollutants from water and wastewater, and as an additive in the biological processing of waste. Regarding MSS biochar, current research primarily focuses on conditions for its production and its characterization. In environmental applications, MSS biochar is predominantly utilized as an adsorbent for various inorganic and organic pollutants. The environmental use of MSS biochar can be limited by its pollutant content and the associated toxicity.

To expand the applications of MSS biochar, such as its use as a soil amendment or in soil remediation, modifications to the MSS biochar are necessary. The incorporation of co-substrates into the pyrolysis of MSS is a suitable option for upgrading MSS biochar properties and reducing or eliminating its potential toxicity before applying it to the soil. Co-pyrolysis combines two or more feedstocks in a single pyrolysis system, even if they are vastly different. However, choosing the right co-substrate and optimizing the co-pyrolysis process are crucial factors for minimizing the negative effects of biochar application to soil [16,17].

The variety of biomass and waste materials available as potential co-substrates for biochar production via co-pyrolysis of MSS has led to a growing number of investigations in this field. Consequently, it is an opportune moment to summarize the effects of different co-substrates on biochar properties because, since 2023, the few reviews specifically dedicated to MSS co-pyrolysis have not focused on these effects. For example, Mohamed et al. concentrated on factors influencing the stabilization of heavy metals (HMs) in biochar, elucidating the mechanisms involved in this process and addressing toxicity reduction in biochar by the co-pyrolysis of MSS and biomass [17]. Likewise, Fan et al. focused on the catalytic and synergistic effects of both inorganic and organic additives on biochar functionality and HM immobilization within the context of co-pyrolysis [18].

This review consists of two parts. Part 1 addresses gaps in the literature by focusing on the comprehensive characterization of co-substrates used in MSS co-pyrolysis and biochar production, sourced from both conventional (biomass) and unconventional (non-biomass) origins. It highlights the importance of selecting appropriate co-substrates and adjusting pyrolysis conditions, which are crucial for enhancing the quality of the resultant biochar. Part 2 examines the effects of co-substrates from different origins on the physicochemical and chemical properties of MSS biochar. Furthermore, this section considers the broader environmental implications of using co-pyrolyzed biochar, including an analysis of environmental risks associated with HMs and selected emerging pollutants. Finally, it provides an update on the current status of the application of MSS biochar derived from co-pyrolysis for the remediation of HM-contaminated soils. By exploring various co-substrates and their effects on biochar properties, this review aims to develop optimized pyrolysis strategies to produce high-quality biochar with enhanced environmental benefits. This comprehensive review addresses both the technical and environmental aspects of MSS biochar production and its enhancement through co-pyrolysis, highlighting its significant potential in sustainable waste management and soil remediation.

For this review, we conducted a systematic search of major scientific databases such as Web of Science, Scopus, and ScienceDirect using specific keywords such as “sewage sludge pyrolysis”, “co-pyrolysis”, and “biochar” to find relevant literature. We prioritized recent publications (5–10 years) and focused on experimental studies, review papers, and theoretical papers addressing different aspects of MSS pyrolysis and co-pyrolysis. We also cross-referenced relevant review articles and meta-analyses to ensure comprehensive coverage of the topic.

## 2. MSS as Feedstock for Biochar Production

MSS is a mixture of water, organic matter (including microorganisms and decayed organic debris, polysaccharides, lipids, proteins, plant macromolecules, and some micropollutants), and inorganic components such as carbonates, phosphates, sulfides, and predominantly non-crystalline oxides and hydroxides of iron, aluminum, and manganese that can originate from soil and some synthetic polymers [19]. MSS is rich in organic matter, the content of which can range widely from 25% to 90%, depending on the technology of MSS stabilization [20]. Raw MSS consists mainly of a bacterial mass composed of fats, protein, cellulose, organic acids, and humic substances. Aerobic or anaerobic stabilization reduces the organic content and alters the compounds. Stabilized MSS contains stable organic compounds like microbial tissue, lignin, cellulose, lipids, organic-N compounds, and humic-like materials [20]. In addition, MSS is a source of phosphorous (P) (up to 2.5%), nitrogen (N) (up to 9%) [19], and potassium (K) (up to 1.1%) [21].

The production of MSS varies depending on the type of WWTP and its process configuration. MSS can be produced during primary treatment (involving physical and/or chemical processes), secondary treatment (biological processes), and tertiary treatment (additional to secondary treatment, often involving nutrient removal). In a conventional WWTP, the majority of MSS originates from primary sedimentation tanks, where organic and inorganic materials from raw wastewater settle, and from secondary sedimentation tanks following biological treatment. The composition of this MSS is influenced by the characteristics of the wastewater source and the operation of the primary sedimentation tanks [22,23].

Several factors contribute to the composition and properties of MSS. These factors include the origin of the wastewater, the purification treatment undergone by the wastewater, the treatment for stabilizing the MSS, the duration and conditions of MSS storage, and the use of coagulant agents. It is crucial to standardize treatments within WWTPs. Even when employing the same treatment methods, slight variations in the conditions, such as the type of flocculation agent or the temperature of stabilization, can lead to alterations in MSS composition.

MSS also contains pathogens and potentially toxic inorganic and organic compounds, posing significant environmental and public health concerns. These pollutants mainly originate from various types of wastewater entering WWTPs, including domestic and industrial wastewater, as well as urban runoff, with the latter having the least impact on HM levels in MSS.

**Figure 3:**
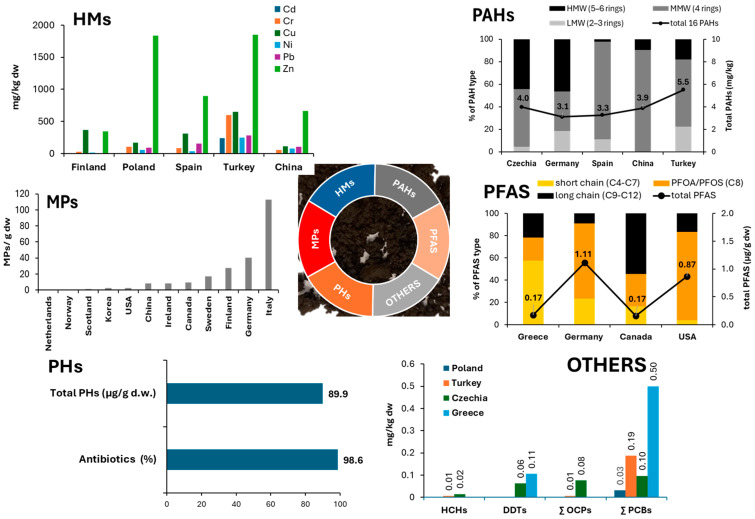
Types of pollutants in MSS (heavy metals, HMs; polycyclic aromatic hydrocarbons, PAHs; microplastics, MPs; per- and polyfluoroalkyl substances, PFASs; pharmaceuticals, PHs; Others; hexachlorocyclohexanes, HCHs; dichlorodiphenyltrichloroethane, DDTs; organochlorine pesticides, OCPs; polychlorinated biphenyls, PCBs) in MSS from different countries. HMW—high molecular weight, MMW—medium molecular weight, LMW—low molecular weight [14,31,32,33,34,35,36,37,38,39,40,41,42,43,44,45,46,47,48,49,50,51,52,53,54].

Figure 3 presents the content of HMs and organic pollutants in MSS from selected countries. HMs are commonly found in MSS. HM transfer largely depends on the type of MSS and is controlled by physical and biological processes. The total HM concentration in MSS can vary considerably depending on the source of the wastewater and the wastewater treatment technologies. The content of HMs in MSS from different WWTPs typically decreases in this order: Zn > Pb > Cu > Cr > Ni > Cd (Figure 3). The total amount of HMs in MSS does not fully reflect their ecological impact. HM mobility and bioavailability are crucial for predicting their release from MSS and transfer to the environment. HM behavior in MSS also depends on the properties of the MSS (e.g., pH, electrical conductivity, potentially mineralizable nitrogen, and ionic strength) [24,25,26].

**Figure 4:**
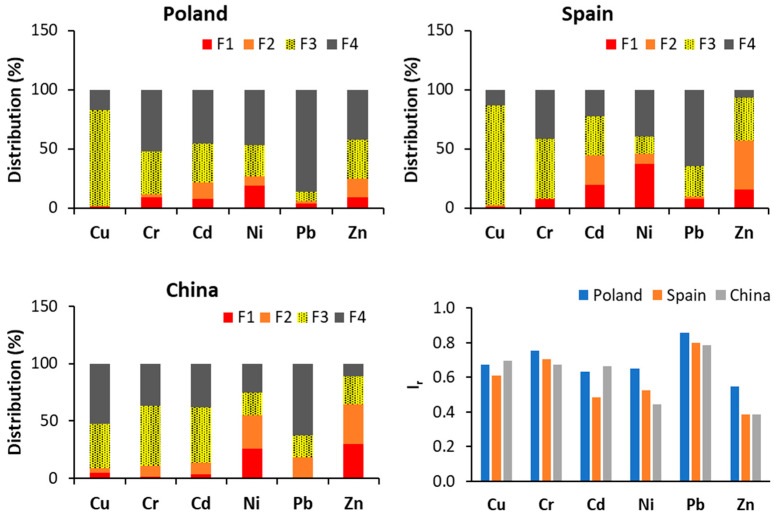
Distribution and stability (as I_r_ index) of HMs in MSS from selected countries (F1 is the exchangeable and acid soluble fraction, corresponding to high HM mobility; F2 is the reducible fraction, moderate HM mobility; F3 is the organic fraction, moderate HM mobility; F4 is the residual fraction, low HM mobility). For the I_r_ index, the higher the value (which ranges from 0 to 1), the higher the stability of the HMs [14,31,32,33,34,35,36,37,38,39,40,41,42,43,44,45,46,47,48,49,50,51,52,53,54].

Figure 4 shows HM distribution in MSS. Based on their fractionation, especially on HMs’ share in exchangeable and acid soluble fraction and reducible fraction, their mobility exhibits varying degrees, depending on HM type. Based on HM fractionation in MSS from WWTPs from different countries, the most mobile are Cd, Cr, Ni, and Zn. The higher the share of HMs in F1 and F2 fraction, the lower the HM stability in MSS, which reflects values of reduced partition index (I_r_) that are used for comparison of HM stability in different solid matrices.

In recent years, there has been a growing emphasis on identifying organic pollutants, particularly emerging contaminants, in MSS [27,28,29,30]. The most concerning organic pollutants found in MSS include polyaromatic hydrocarbons (PAHs), polychlorinated biphenyls (PCBs), polychlorodibenzo-p-dioxins and polychorodibenzofurans (PCDD/Fs), polybrominated diphenyl ethers (PBDEs), phthalates (PAEs), and perfluorinated compounds (PFCs). Additionally, there are emerging concerns about the presence of pharmaceuticals, especially antibiotics and personal care products (PPCPs) (Figure 3). Thus, when MSS is applied to land, it could potentially increase the levels of micropollutants in soil, runoff, and groundwater.

Treating wastewater and MSS can result in the formation of intermediate compounds, which can be more hazardous than the original pollutants. In a two-year investigation by Bueno et al. on five WWTPs, a persistent group of 100 organic compounds belonging to various chemical groups, such as pharmaceuticals, personal care products, pesticides, and metabolites, was identified [55]. Meanwhile, Clarke et al. conducted a critical review and placed a list of contaminants of concern in order of priority [30]. These include perfluorinated chemicals (PFOS, PFOA), polychlorinated alkanes (PCAs), polychlorinated naphthalenes (PCNs), organotins (OTs), polybrominated diphenyl ethers (PBDEs), triclosan (TCS), triclocarban (TCC), benzothiazoles, antibiotics and pharmaceuticals, synthetic musks, bisphenol A, quaternary ammonium compounds (QACs), steroids, phthalate acid esters (PAEs), and polydimethylsiloxanes (PDMs).

Other contaminants found in municipal wastewater, such as per- and polyfluorinated substances (PFASs) and microplastics (MPs), have also been detected in MSS. PFAS compounds with higher hydrophobicity tend to bind more strongly to the organic matter present in MSS during the wastewater treatment process. Studies indicate that WWTPs effectively reduce the concentration of MPs from influent to effluent streams. However, the degree of reduction varies depending on the specific treatment used, ranging from 58.8% to 99.9% around the world [56,57,58]. Nonetheless, a portion of MPs may still accumulate in MSS. Population does not seem to be a suitable predictor of MP levels in MSS. As can be seen in Figure 3, Italy exhibited the highest levels of MPs in MSS, while the Netherlands showed the lowest levels, even though the population density in the Netherlands is significantly higher. Moreover, although China generates the highest annual volume of MSS, the average reported MP content in MSS was relatively low.

It is a well-established fact that there is a direct relationship between the concentration of pollutants in MSS and population density. A study conducted by Wojciula et al. revealed that the meta-stable fraction of Pb in MSS increases with an increase in population equivalent [59]. Similarly, Košnář et al. found that there is a linear correlation between the size of the WWTP, which is directly related to the population equivalent, and the concentration of persistent organic pollutants (POPs) in the MSS [45]. Therefore, the available evidence suggests that as the population equivalent increases, the concentration of certain pollutants in the resulting MSS, such as HMs and POPs, also tends to increase. This can be important when selecting MSS for biochar production with the aim of reducing environmental risk.

According to the European Biochar Certification (EBC), biochar can be produced from various sources, including conventional (agriculture, forestry) and industrial (textile, anaerobic digestion, wastewater treatment) ones (Table 2).

The type of biomass strongly influences the quality of biochar and its intended application. The EBC defines seven certification classes of biochar: FeedPlus, Feed, AgroOrganic, Agro, Urban, Consumer Materials, and Basic Materials [61]. The different classes of biochar are suitable for certain applications: FeedPlus and Feed biochars comply with EU and European Free Trade Association (EFTA) regulations and are suitable for livestock and soil applications. Agro and AgroOrganic biochar comply with EU fertilizer regulations and are ideal for urban soil application. Urban biochar is suitable for non-food soil application, e.g., for tree planting and park maintenance. The Consumer Materials class is suitable for consumer or food products. Finally, Basic Materials biochar is suitable for major industries such as construction [61].

To eliminate hazards when producing EBC-class biochars, guidelines for pollutant content in biomass feedstock and pyrolysis conditions must be followed. For instance, plastic and rubber waste should not exceed 1% (m/m) or can be up to 10%, respectively (Consumer and Basic Materials). When using primary agricultural products, soil organic carbon preservation is crucial. Animal byproducts can be pyrolyzed for all certification classes except for FeedPlus and Feed biochars. The pyrolysis temperature should be above 500 °C to eliminate hazards and micropollutants.

Taking into account the complex composition of MSS, which includes inorganic and organic compounds, nutrients, microorganisms, and pollutants [2], the EBC restricts the use of MSS as biomass for pyrolysis and the use of MSS biochar in agriculture, as the removal of pollutants from it is uncertain. The EBC recommends classifying MSS biochar in the Basic Materials class, as indicated in Table 2. However, MSS could be reincluded in the list of permitted feedstocks for pyrolysis if solid and comprehensive technical and scientific evidence demonstrates the safe use of materials produced by MSS pyrolysis. If this could be accomplished, there would be considerable potential for the use of MSS biochar in soil remediation. It is important to note that MSS biochar is approved for use in agriculture in some countries, such as Czechia, Sweden, Italy, Denmark, Estonia, the U.K., Norway, Israel, and Australia. Nevertheless, the challenge is to improve the quality of MSS biochar to make it acceptable for such applications according to EBC regulations.

## 3. MSS Pyrolysis and Co-Pyrolysis

The MSS pyrolysis is a multistage process during which a variety of gases, such as H_2_O, CO_2_, CO, H_2_, and CH_4_, as well as non-condensable hydrocarbons and condensable organic compounds like tar, are produced [62]. Generally, the flammable H_2_ and CO gas tend to become more abundant as the pyrolysis temperature increases, while CO_2_ content tends to decrease, and CH_4_ content may fluctuate with the temperature rise. However, the reactor arrangement and other operational parameters can strongly influence the specific behaviors of these compounds [63,64,65].

Based on thermogravimetric and derivative thermogravimetric analysis, MSS pyrolysis can be divided into three main stages: dehydration, depolymerization, and complete degradation [66]. The process begins with transferring heat to the surface of the MSS particle through radiation and/or convection, which gradually spreads to the interior. In the first stage (<200 °C), the mass loss (mostly few %) is due to dehydration of the MSS and removal of free and chemically bonded water. The main product during this stage is water vapor, with small quantities of CO_2_ and CH_4_ due to the decomposition of proteins, carbohydrates, and lipids present in the MSS. Steam vapors in further stages of pyrolysis can improve H_2_ production. During the second phase (200–350 °C), a considerable mass loss of MSS (up to 70%) is observed due to the breaking of carbon–carbon (C-C) bonds within the carbonaceous components of the MSS. Apart from CO_2_, other gaseous products (CH_4_, H_2_, carboxylic acids) are present, indicating depolymerization reactions. In addition, large molecular intermediate fragments are generated. With a further increase in temperature (350–550 °C), the secondary decomposition of biochar occurs, and hydrocarbons and aromatic compounds in the volatile phase are produced. As a result, there is a decrease in combustible carbon, water vapor, and CH_4_ with an increase in H_2_, CO, and CO_2_ contents. Finally, at the last stage (550–900 °C), there is a complete pyrolysis. The weight loss is much lower (by several %) compared to the previous stage due to the decomposition of non-biodegradable substances or resulting from reductive reaction [67]. The contents of H_2_ and CO are high and stable. The carbon residue remains constant due to the formation of biochar through carbonization.

The pyrolysis of MSS into biochar offers numerous benefits, including environmentally safe disposal by reducing MSS volume, eliminating pathogenic bacteria, decomposing organic matter and certain pollutants, and recovering nutrients such as K, P, calcium (Ca), and magnesium (Mg). Additionally, it contributes to climate change mitigation [68]. Nevertheless, biochar obtained solely by pyrolysis of MSS has low C content with low porous structure and high HM content [69], which can limit its practical application in different fields, including soil remediation. One way to reduce the HM content is to mix the MSS with co-substrates that have a lower level of pollutants. Co-pyrolysis offers a wide range of potential in terms of increasing biochar quality by processing two or more feedstocks under the same operating conditions [70]. This not only helps to dilute the amount of inorganic matter and toxic compounds in the MSS but also enhances the physicochemical properties of the resultant biochar, including pore structure, pH, surface functional groups, and mineral components that are important for HM immobilization in soil [71,72,73]. Additionally, co-pyrolysis can help to maintain a high level of nutrients.

The mechanism of co-pyrolysis is similar to that of pyrolysis of MSS alone. Recent studies indicated that compared with biochar derived from MSS alone, biochar derived from co-pyrolysis of MSS and other feedstock, such as crop straw, could reduce the ecological risk mostly in relation to HMs by forming more stable HM fractions and improve physicochemical properties, such as porous surface morphology structure and large specific surface area [71,73,74]. Several studies have examined the impact of varying the mixing ratio of co-substrate materials on the HM content and toxicity of biochar produced from MSS singular pyrolysis. One key finding is that the choice of co-substrate material can play a significant role in determining the quality of the resultant biochar [75,76]. For example, a study by Li et al. showed that the co-pyrolysis of MSS with pine sawdust could enhance the aromatization of biochar, which seems a suitable strategy for better removal of HMs from the medium [77]. Other studies have shown that adding certain amendments, such as Ca or K, can improve biochar quality by reducing HM leaching into surrounding soils [28,78].

Overall, the properties of biochar derived from MSS can be influenced by several factors, including the properties of MSS and co-substrates, as well as the operational conditions of pyrolysis. These factors can all play a role in shaping the physical and chemical properties of the resulting biochar and can impact its potential uses and applications [79,80,81]. For MSS co-pyrolysis, careful consideration of the biomass-to-MSS ratio and temperature is necessary in order to effectively manage pollutant content in MSS biochar and produce high-quality biochar [17,73,82,83].

### 3.1. Types of Co-Substrates Used for MSS Co-Pyrolysis

The process of MSS co-pyrolysis involves a diverse range of co-substrates, including wheat straw, cotton stalk, and rice husk, which have been extensively studied to determine their efficacy. In the current review, there has been presented the use of different types of biomasses as feedstock, which can be broadly classified into six main sources: forest and wood processing, agriculture, food processing residue, recycling economy, anaerobic digestion, and other miscellaneous sources with reference to EBC as guidelines. The process for land application of biochar has strict risk management and safety requirements. To minimize unforeseen risks, researchers rarely experiment with chemicals or waste. As a result, biomass co-substrates have become the most common in MSS co-pyrolysis [18].

**Figure 5:**
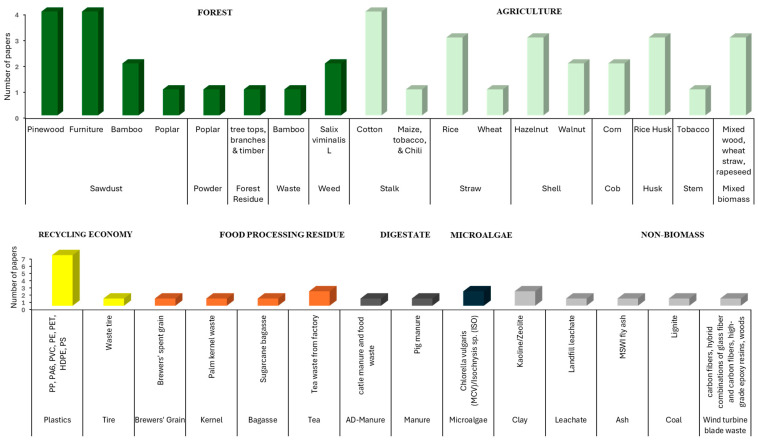
The main categories and types of co-substrates for MSS co-pyrolysis (based on papers published between 2009 and 2024) [73,76,84,85,86,87,88,89,90,91,92,93,94,95,96,97,98,99,100,101,102,103,104,105,106,107,108,109,110,111,112,113,114,115,116,117,118,119,120,121,122,123,124,125,126,127,128,129,130,131,132,133,134,135,136,137,138,139,140,141,142].

Figure 5 provides a detailed overview of recent studies of feedstocks used in co-pyrolysis with MSS. The data from over 60 papers analyzed show that a significant proportion of the feedstocks used for MSS co-pyrolysis come from agriculture. There are many types of agricultural wastes that can be used for MSS co-pyrolysis in the form of stalks, straw, stems, shells, cobs, manure, bagasse, and mixed biomass. Based on the dataset collected, cotton stalk is the predominant MSS co-substrate. Other suitable alternatives from agriculture co-substrate for co-pyrolysis include rice straw, rice husk, and hazelnut shell. The primary components of agriculture biomass, similar to forest biomass, are lignin, cellulose, and hemicellulose. In contrast, MSS primarily contains proteins, fats, cellulose, and carbohydrates [81]. Therefore, agricultural biomass contains more C and much lower HM content than MSS, and this type of biomass can contribute to high specific surface areas, optimized pore structures, and reductions in the total HM contents of biochars when used in co-pyrolysis [82].

Forest biomass can be a suitable co-substrate in terms of biochar production and its characteristics due to high lignin content and low ash content [84]. Forestry operations generate forest residues, treetops, and branches from timber harvesting as well as sawdust. The amount of forest residues can be even a few times higher than MSS [92]. Small-diameter woody fractions typically exhibit a naturally dried moisture content of 38–48% (wet basis) due to natural drying in the forest. With their high heating value (>20 MJ kg^−1^ dry mass), low N (<0.5% dry mass), and low ash (<2% dry mass) content, these fractions complement the higher values for these properties found in MSS. This makes mixtures of MSS and forest waste an interesting option for improving feedstock properties in co-pyrolysis [92]. Based on data in Figure 5, pinewood and furniture sawdust are the most feasible co-substrates for MSS co-pyrolysis. However, it is worth noting that the variability of feedstocks obtained from forests for MSS co-pyrolysis is limited compared to agriculture, although sawdust from the forest has shown promising results and is often used as a feedstock in MSS co-pyrolysis.

Among the recycling economy group of substrates for pyrolysis, there are plastic wastes that pose a significant challenge due to their persistence in the environment if they are not properly discarded or recycled. Research on plastic pyrolysis has been primarily focused on the conversion of plastic into pyrolysis oil and pyrolysis gas [143]. The newest insights into the co-pyrolysis of MSS and plastics showed their beneficial effect on biochar properties. The presence of plastics enhanced the C structure of biochar, the pore structure, and the surface properties of biochar that can be crucial for HM immobilization [124,144]. The PVC can enrich MSS biochar in Ca or Mg due to the presence of CaCO_3_ and Mg(OH)_2_ additives in PVC. This can facilitate HM fixation in the resultant biochar. Moreover, the MSS modified with MPs can reduce the toxicity of Cr and Pb in soil, making these MPs increasingly viable as co-substrate for co-pyrolysis [144]. On the other hand, co-pyrolysis of MSS with waste tires offers a solution to overcome the low calorific value of MSS and optimize the physical and chemical properties of MSS biochar. The resultant co-pyrolyzed biochar may serve as a more suitable and effective adsorbent compared to biochar derived solely from MSS [123].

Microalgae (MA) are promising feedstock for biofuel production due to their high photosynthetic efficiency, high oil content, and rapid growth rate [145,146]. The primary components of MA biomass are proteins, carbohydrates, and lipids [146]. Chlorella vulgaris, a common MA for MSS co-pyrolysis, has 41.51%, 20.99%, and 15.67% of proteins, carbohydrates, and lipids, respectively [147].

In addition to biomass co-substrates, other types of non-biomass co-substrates, such as clay, leachates, fly ash, lignite, and mixed fibers, have also been used in MSS co-pyrolysis. The use of these feedstocks provides a more comprehensive and diverse approach to co-pyrolysis, allowing for greater flexibility, adaptability, and optimization of the process and biochar properties.

### 3.2. Composition of Substrates Used in Co-Pyrolysis With MSS

Feedstock composition is an important determinant of the composition and yield of pyrolysis products. Differences in composition between MSS and other co-substrates biomass result in variations in the behavior of pyrolysis and obtained final biochar. In contrast to MSS, biomass, as the most important co-substrate, has a lower moisture content and a higher carbon content than MSS. It consists mainly of cellulose, hemicellulose, and lignin, with a lower nitrogen (N) and sulfur (S) content. It is pertinent to note that specific agriculture or forest residues many contain high levels of N and/or S. The main effects of a particular feedstock on biochar, depending on the characteristics of the feedstock, are summarized in Table 3.

In general, feedstocks with low moisture, VM, ash, and oxygen content, high FC content, high lignin content, and smaller particle size result in higher biochar yields during pyrolysis. To optimize the pyrolysis process and maximize the yield of each product, including biochar, a thorough understanding of the effects of feedstock properties is essential. Therefore, proximate, ultimate, and biochemical analyses are crucial methods for analyzing the composition of co-substrates for co-pyrolysis with MSS.

#### 3.2.1. Proximate Analysis

The proximate analysis includes moisture, VM, ash, and FC contents. A low moisture content in the feedstock reduces energy consumption during pyrolysis and often lowers the heating rate during the process. For a given feedstock, a moisture level of up to 10% is the most suitable for pyrolysis [148]. Otherwise, some feedstock pre-treatment (e.g., dewatering, drying) is necessary.

**Figure 6:**
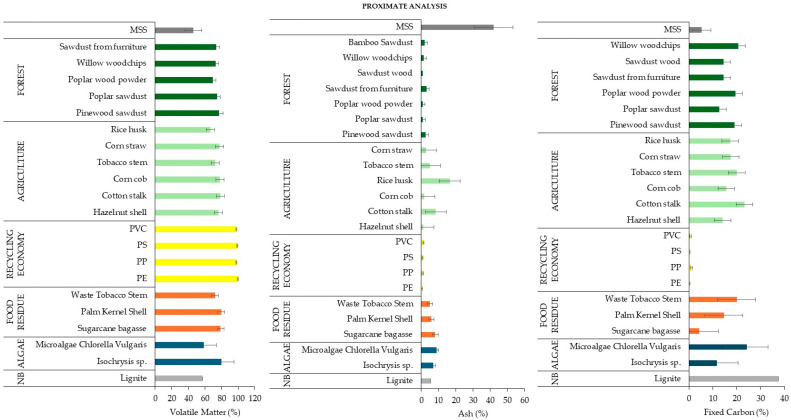
The results of proximate analysis of MSS and co-substrates based on different studies (as median values with error bars of standard deviation) [84,87,88,93,95,100,101,102,104,106,111,122,130,132,135,136,151]; NB means non-biomass.

For a comprehensive overview of MSS and the diverse co-substrates utilized in co-pyrolysis, Figure 6 provides the average values of proximate analysis for co-substrate used in MSS co-pyrolysis. The content of VM in the combustible organic fraction of MSS is generally lower than in other co-substrates. This is due to the fact that other co-substrates, for instance, those from agriculture, are rich in cellulose, hemicellulose, and lignin, resulting in a higher volatile fraction than in MSS. When comparing the VM content of MSS with that of co-substrates derived from forests, agriculture, and those categorized as non-biomass, the average VM of MSS was significantly lower. MSS had an average VM of 45.9%, while forests and agriculture had 75.8% and 76.6% (wt.%), respectively.

Ash content is a measurement of a material’s non-volatile and non-combustible elements. However, MSS has a significantly higher ash content than other co-substrate categories. The average ash content of MSS is 42.0%, while the other categories have values of less than 7.9%. This difference is mainly due to the inorganic minerals that remain as ash after combustion. Other co-substrates generally have a lower inorganic content, resulting in a lower ash content compared to MSS. However, it is important to note that the inorganic and ash content of co-substrates can vary considerably depending on their origin and type.

A high FC content in biomass is usually associated with a high lignin content. Biomass rich in lignin generally provides higher yields of biochar than biomass rich in cellulose and hemicellulose. The FC content of MSS is lower than that of forestry and agricultural substrates but higher than that of substrates categorized as “circular economy”, suggesting an inverse relationship between FC content and ash content. Considering that the average ash content of MSS is significantly higher than that of other substrates, one would expect the FC content to be lower.

#### 3.2.2. Ultimate Analysis

The ultimate analysis of the feedstock (carbon, hydrogen, oxygen, nitrogen, and sulfur), as well as the H/C and O/C ratios, is crucial for maximizing biochar yield during pyrolysis or co-pyrolysis. Lower H/C and O/C ratios in the feedstock are preferred for higher biochar yields. Therefore, feedstocks with higher C content produce more stable biochar with increased yield. Lower nitrogen and sulfur content in the feedstock are preferred to ensure biochar purity and increase biochar yield [146]. Biomass materials intended for the production of high-quality bio-oil in high yields via co-pyrolysis with MSS should have a high H/C ratio, a low O/C ratio, and low oxygen, ash, nitrogen, and sulfur contents [146]. Plastic waste, for example, usually has a high H/C ratio and a low O/C ratio, which leads to the production of more hydrocarbon byproducts during pyrolysis [149,150].

**Figure 7:**
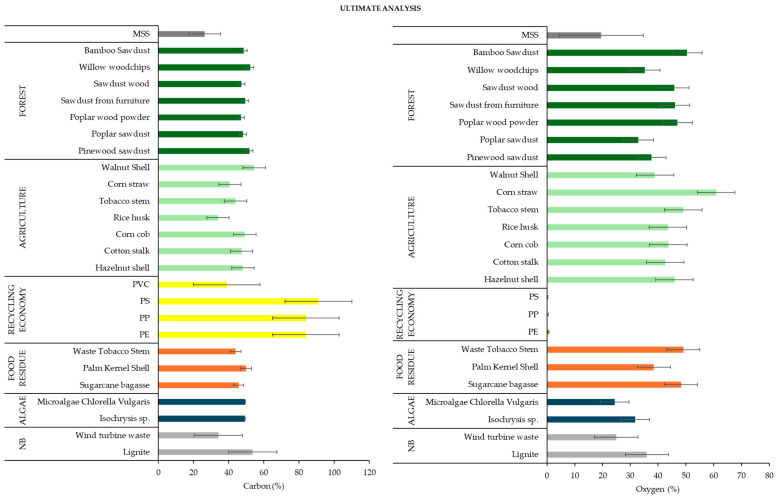

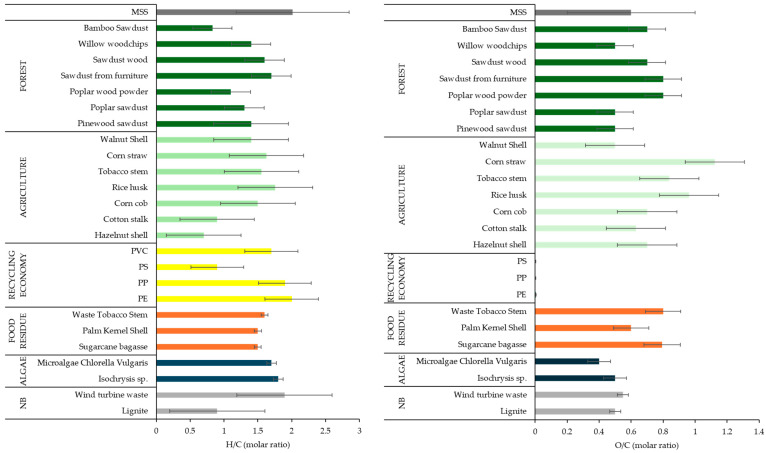
The results of ultimate analysis of MSS and co-substrates based on different studies (as median values with error bars of standard deviation) [84,87,88,93,95,100,101,102,104,106,111,122,130,132,135,136,151]; NB means non-biomass.

The analysis of ultimate, and H/C trends in MSS and other co-substrates revealed that the C content of the co-substrates was found to be much higher than that of the MSS (Figure 7). The forest, agriculture, and food residue co-substrates recorded average C contents of 48.5%, 44.3%, and 45.6%, respectively, while the MSS had a carbon content of 26.4% (wt.%).

Lignin is a complex macromolecule that possesses a distinctive three-dimensional structure that comprises various types of chemical bonds. In contrast to hemicellulose and cellulose, the degradation of lignin does not occur within a specific temperature range.

As far as the molar H/C ratio is concerned, the MSS showed significantly higher values than the other co-substrates. The average molar H/C ratio was 2.0 for the MSS, compared to 1.4 for the forest and 1.5 for the agricultural co-substrates. When comparing the molar O/C ratios, it was found that maize straw of agricultural origin had the highest value for the molar ratio at 1.0. This was followed by poplar wood powder, sawdust from furniture, and tobacco stalk waste, with a value of 0.8.

Overall, the data indicate that the VM and C content in MSS are lower than those of other co-substrates. However, it is notable that the MSS exhibits a significantly higher ash content and H/C molar ratio than other co-substrate groups.

#### 3.2.3. Biochemical Analysis

Biomass obtained from plant residues, specifically that derived from forests and agriculture, contains three main organic compounds, namely cellulose, hemicellulose, and lignin. Under thermal treatment, these compounds exhibit distinct behaviors; hence, the composition of biomass plays a crucial role in determining product yield and quality [152]. Hemicellulose, a class of polysaccharides with a branched-chain structure, is the most reactive of these compounds. Hemicellulose decomposes at temperatures ranging from 220 to 315 °C. During the process of torrefaction, the primary mechanism of biomass degradation is the breakdown of hemicellulose. However, this process is highly sensitive and presents challenges in terms of control since the most significant changes in properties occur within a narrow temperature range during hemicellulose decomposition. In contrast to hemicellulose, cellulose is a polysaccharide that lacks branching. It exhibits greater thermal stability and decomposes at temperatures ranging from 280 to 400 °C [152,153].

This is because the compound contains various functional groups with different thermal stabilities, causing it to decompose over an extensive temperature range. The thermal breakdown of lignin initiates at approximately 200 °C, and temperatures up to 900 °C (depending on the residence time) may be necessary for complete decomposition [152].

**Figure 8:**
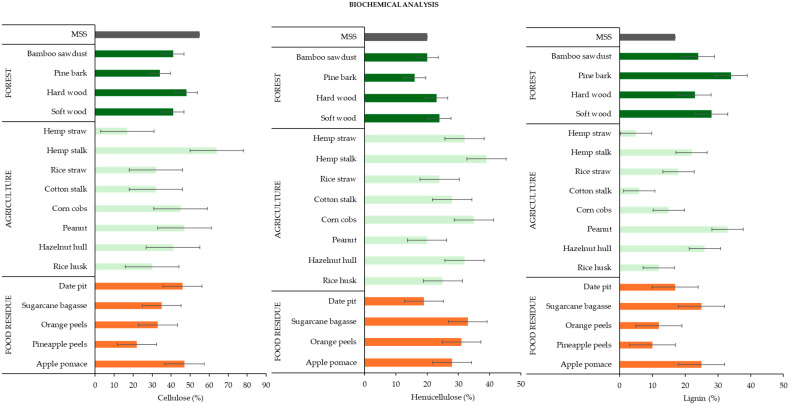
The results of biochemical analysis of MSS and co-substrates based on different studies (as median values with error bars of standard deviation) [121,156,157,158,159,160,161,162,163,164,165,166,167,168,169].

Figure 8 illustrates a comprehensive comparison of the cellulose, hemicellulose, and lignin content in different co-substrates. Cellulose, the primary component of biomass, facilitates synergistic effects during MSS co-pyrolysis [121]. Hemicellulose decomposition at lower temperatures generates volatiles that can catalyze the pyrolysis of MSS [154]. The thermally stable aromatic structure of lignin acts as a radical initiator, promoting the cracking of larger molecules present in MSS during co-pyrolysis [155]. Notably, hemp stalks from the agriculture group exhibit a higher content of lignocellulose than hemp straw. This implies that incorporating hemp stalk with MSS will yield superior biochar compared to other feedstocks. Nevertheless, other feedstocks also showed promising compositions of all three organic compounds, such as apple pomace, sugarcane bagasse, and hazelnut shell.

#### 3.2.4. Particle Size and Density

The size of MSS and co-substrate particles is a significant factor in the process of biomass decomposition. When the particles are large, they undergo incomplete decomposition, which ultimately leads to the formation of biochar. Conversely, small particles promote additional surface reactions, which can speed up the process of biomass decomposition and increase heating rates [170]. As the particle size increases, the temperature gradients within the material particles also increase. When the core of the biomass particle is carbonized but not fully decomposed, the temperature gradient causes the core temperature to be lower than the surface temperature. As a result, biochar production increases while the production of liquid and gas products decreases [171,172]. Although there is no explicit particle size for MSS and co-substrates pyrolysis, studies have shown that particle size < 5 mm is commonly used.

### 3.3. Conditions of MSS Pyrolysis and Co-Pyrolysis

**Figure 9:**
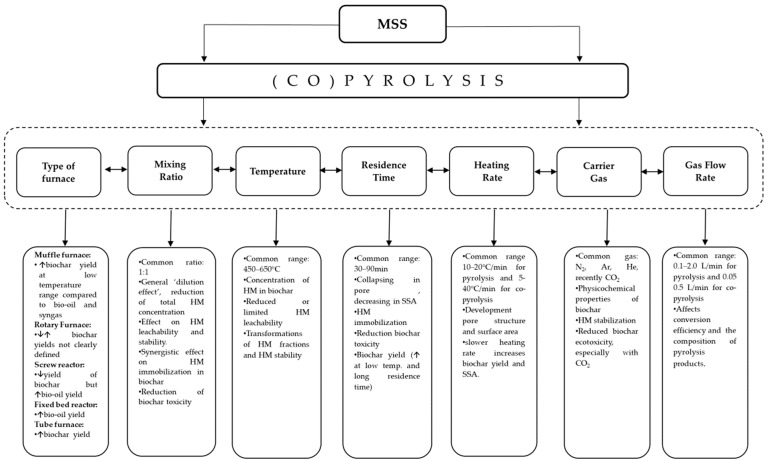
Summary of the main operational conditions of pyrolysis and co-pyrolysis and their effects on MSS biochar. ↑ increase; ↓ decrease; ↓↑ ambiguous.

The conditions of a pyrolysis or co-pyrolysis process play essential roles in determining the yield, quality, and properties of the resulting product. These specific conditions include the type of furnace as well as operational conditions, such as mixing ratio (only for co-pyrolysis), temperature, residence time, heating rate, carrier gas, and gas flow rate (for pyrolysis and co-pyrolysis), which are instrumental in ensuring the desired outcome. As such, the optimization of these conditions requires careful consideration and attention. Figure 9 summarizes the main effects of pyrolysis conditions on MSS biochar, while Table 4 provides a comprehensive analysis of the effects of operational conditions of pyrolysis and co-pyrolysis on MSS biochar yield, depending on the furnace type.

#### 3.3.1. Type of Furnace

One of the most important pieces of equipment for pyrolyzing feedstock is a pyrolysis furnace, which primarily converts complex organic molecules into simpler molecules [173] by providing enough heat energy to pyrolyze raw materials at high temperatures [174]. For MSS pyrolysis and co-pyrolysis at the laboratory scale, several types of furnaces have been used: muffle furnaces [155], rotary furnaces [175], screw reactors [93], fixed-bed reactors [176], microwave pyrolysis reactors [177,178], and tube furnace [179]. The review focuses exclusively on biochar derived from MSS pyrolysis and co-pyrolysis, thus excluding studies that primarily investigate bio-oil and syngas production using the same reactors. Table 4 presents studies that emphasize biochar production from MSS pyrolysis or co-pyrolysis, providing a comparative scope of the research.

Muffle furnaces are so named because the heating elements are placed outside the furnace chamber, and the material to be heated is placed inside a separate chamber called the muffle. Typically, the muffle is made of a ceramic material that can withstand high temperatures and is resistant to chemical corrosion. These furnaces work on the principle of indirect heating. When the furnace is turned on, an electric current passes through the heating elements, which heat up and radiate heat toward the muffle, which in turn absorbs the heat and becomes hot, heating the material inside.

The main parts of a muffle furnace consist of the heating elements, muffle, temperature controller, thermocouple, power supply, insulating, exterior casing, and exhaust system [180]. The various kinds of muffle furnaces that are available include the box furnace, tube furnace, vacuum furnace, and box-type furnace [181,182].

In muffle furnace pyrolysis, the mass of feedstock that is utilized varies according to the configuration and needs. For example, vacuum muffle furnaces manufactured by SH Scientific have holding capacities ranging from 1.5 L to 31 L. Customization is also possible, as a 137 L furnace was made for a client [183]. de Oliveira Paiva et al. used a Jung (N1200) muffle furnace to make biochar from 150 to 320 g of material, depending on the kind and bulk density of the feedstock [184]. Muffle furnaces have the advantage of being able to reach high temperatures, usually up to 1200 °C, which makes them perfect for calcination, sintering, and annealing [180]. However, they suffer from the disadvantages of low heating efficiency and excessive energy consumption [185]. The properties of MSS biochar from a muffle furnace can differ from that produced in other furnaces, mainly because oxygen is present in muffle furnaces at the beginning of pyrolysis, and the temperature can vary more in these furnaces (±5 °C) than in electric tube furnaces (±1 °C) [186].

A rotary furnace is a barrel-shaped furnace that is rotated around its axis during heat treatment. It is tilted slightly to allow the sample to be passed from one end of the barrel to the other. In a rotary furnace, hot gases are passed through the chamber to perform heat treatment. To protect the steel body of these furnaces from the extremely high temperatures that are generated inside them and to prevent corrosion, refractory materials are typically used for the lining. Depending on the requirements of the application, bricks, cement, or moldable refractory materials can be used. These materials are generally capable of withstanding temperatures as high as 1000 °C [187].

Unlike rotary furnaces, screw reactors are stationary and use a screw conveyor to move the feedstock. This means that the reactor uses mechanical drives to move the biomass, resulting in reduced residence times, increased control, and improved fine feedstock mixing. The reactor’s exterior walls can be heated, and the reactor can supply heat by adding hot sand or ceramic balls to the biomass [188,189]. The typical temperature range for screw reactors used in pyrolysis applications is usually between 300 and 900 °C, with the most common range being 400–500 °C [190], so the high transfer coefficients make it very effective for large-scale applications [191]. These reactors are often used for MSS pyrolysis/co-pyrolysis focusing on bio-oil production as compared to biochar yield [186], and they have a simple, reliable design with suitable temperature control. They are suitable for processing large particle sizes (up to 3–5 cm) without the need for carrier gas. However, the drawback of the screw reactor is that the material sticks or is heated unevenly due to localized overheating around the screw [192].

**Table 4:** The effect of furnace type and operational conditions of (co)pyrolysis on MSS biochar yield based on different studies.

Feedstock/Mixing Ratio			Temp. (°C)	HR (°C/min)	RT (min)	CG	FR (L/min)	BY (%)	Research Aim		Ref.
**Muffle Furnace**											
**Pyrolysis**											
MSS			350	10	120	nr	nr	88.3	The impact of pyrolysis temperature on biochar characteristics		[193]
			400					ca. 84			
			450					ca. 82			
			500					64.7			
			200	10	120	nr	nr	92.2	The impact of biochar produced at different temperatures on urban soil fertility and turf grass growth		[194]
			300					81.7			
			500					67.8			
			700					65.1			
			300, 400, 500	11	30	nr	nr	nr	The effects of temperature on the agro-chemical and physical properties of biochar		[195]
			300	17	30	N_2_	0.2	62.5	Investigating the feasibility of biochar production from MSS pyrolysis		[196]
			400		30			28.5			
			500		30			27.3			
			300		60			58.1			
			400		60			25.5			
			500		60			27.0			
			300		90			64.2			
			400		90			27.5			
			500		90			31.0			
**Co-pyrolysis**											
	MSS + Reed (RD) (*P. australis*)			10	120	N_2_	0.05		Analysis of P and HM transformations in biochar		[154]
	MSS:RD 75:25 wt.%		300					ca. 61			
			500					ca. 58			
			700					ca. 56			
	MSS:RD 50:50		300					ca. 54			
			500					ca. 49			
			700					ca. 47			
	MSS:RD 25:75		300					ca. 49			
			500					ca. 45			
			700					ca.42			
	MSS + Rice Straw (RS)		400	nr	120	nr	nr		Cd immobilization in paddy soil under biochar amendment		[108]
	MSS:RS 1:3 wt.%							51.5			
	MSS:RS 1:2							54.3			
	MSS:RS 1:1							59.3			
	MSS:RS 2:1							64.0			
	MSS:RS 3:1							66.1			
	MSS + Tea Waste MSS:TW 1:1		300	nr	120	nr	nr	53.2	Cd removal from aqueous solution		[114]
								53.2	Methylene blue removal from aqueous solution		[117]
	MSS + Brewers’ Spent Grain (BSG)			5	120	N_2_	0.05		Ammonia-nitrogen removal from aqueous solution		[138]
	MSS:BSG 8:2 wt.%		400					46.8			
			500					37.4			
			600					35.9			
			700					33.1			
	MSS:BSG 6:4		400					50.1			
			500					42.2			
			600					39.4			
			700					37.3			
	MSS:BSG 4:6		400					54			
			500					49.4			
			600					46.3			
			700					42.5			
	MSS:BSG 2:8		400					59.6			
			500					54.1			
			600					52.4			
			700					49			
**Fixed-Bed Reactor**											
**Pyrolysis**											
MSS			500	20	20	N_2_	0.02	59	Investigating the conversions of MSS-nitrogen into primary product (NH_3_-N, HCN-N, HCN/NH_3_)		[197]
			600					52.4			
			700 800					47.2 45.6			
MSS			700	10	60	N_2_	0.2	38.7	Investigating the impacts of organic and inorganic constituents on pyrolysis products		[198]
**Co-pyrolysis**											
	MSS + Poplar Wood (PW)		800	10, 30	20	N_2_	0.06		Evaluating the synergistic effects of poplar wood co-substrate yield		[85]
	MSS:PW 8:2 wt.%							ca. 59			
	MSS:PW 6:4							ca. 53			
	MSS:PW 4:6							ca. 44			
	MSS:PW 2:8							ca. 35			
	MSS + Pinewood Sawdust		450	30	30	N_2_	0.4	ca. 54	Investigating the synergistic effects of the product yield and distribution		[84]
			500					ca. 53			
			550					ca. 49			
			600					ca. 48			
	MSS + Pine Sawdust		800	40	15	H_2_	nr	nr	Methylene blue removal from aqueous solution		[90]
	MSS + Rice Husk		900	10	120	N_2_	nr	20	Investigating the synergetic effects of co-substrate on gas and biochar production		[118]
	MSS + Microalgae *Isochrysis* sp. (ISO)		500	5, 10, 15, 20, 25	nr	N_2_	0.4		Evaluation of biocrude assessment and biochar yield		[135]
	MSS:ISO 1:1 wt.%							35.3			
	MSS:ISO 1:2							41.4			
	MSS:ISO 2:1							46.2			
	MSS + Lignite		900	10	120	N_2_	nr	24	Analysis of product yields and composition of co-pyrolysis		[136]
**Tube Furnance**											
**Pyrolysis**											
MSS			220	nr	30	N_2_	1.7	91.3	Analysis of the total and available contents of Ca, K, Mg, P, and S in biochar		[179]
			320					70.5			
			420					60.0			
			520					53.2			
			620					50.3			
			500, 700	10	60	N_2_	0.1	nr	Ferrous sulfate modification and treatment on biochar		[199]
			300	12–13	120	N_2_	2	84.4	P removal from aqueous solution		[186]
			400					66.4			
			500					60.5			
			600					58.4			
**Co-pyrolysis**											
	MSS + Bamboo Waste (4:1 mass ratio)		700	10	30	N2	nr	nr	Ciprofloxacin removal from aqueous solutions		[103]
	MSS + Hazelnut Shell (4:1 wt.%)		900	10	90	N_2_	0.3	nr	Analyzing thermal decomposition reaction and interaction of biochar		[100]
	MSS + Walnut Shell (3:1, 1:1, 1:3 mass ratio)		600	10	180	N_2_	0.3	nr	Ammonium and phosphate removal from water		[101]
	MSS + Wheat Straw (WS)			nr	30	N_2_	0.5		Investigating the combustion reactivity of biochar		[105]
	MSS:WS 8:2 wt.%		900					ca. 25			
	MSS:WS 6:4							ca. 20			
	MSS:WS 4:6							ca. 17			
	MSS:WS 2:8							ca. 14			
	MSS + Waste Tire (0:10, 1:9, 3:7 wt.%)		300, 500, 700	10	120	N_2_	0.3	nr	Cd and tetracycline removal from water		[123]
	MSS + PP, PA6, PVC (8:2 wt.%)		800	15	60	N_2_	0.1	nr	Analyzing the release of N, S, and Cl by different plastics share in biochar		[124]
	MSS + Wind Turbine Blade Waste (8:2, 7:3, 4:6, 5:5 wt.%)		600	10	60	N_2_	0.1	nr	Evaluation of wind blade co-substrate for carbon capture		[134]
	MSS + Red Mud and Steel Slag (100:0, 80:20, 60:40, 33:67, 0:100 wt.%)		900	10	120	N_2_	nr	nr	Tetracycline removal from wastewater		[200]
**Microwave Reactor**											
**Pyrolysis**											
MSS			nr	nr	10	N_2_	0.01	nr	Cu removal from aqueous solution		[201]
**Co-pyrolysis**											
	MSS + Cotton Stalk (CS)			nr	30	N_2_	nr		The effect of co-substrate on biochar properties		[177]
	17:3 (MSS:CS) wt.%		450					49.62			
			550					ca. 43			
			650					ca. 41.5			
			750					35.16			
	7:3 (MSS:CS)		450					43.25			
			550					ca. 39.5			
			650					ca. 36.5			
			750					31.41			
			700								
			800								
			900								
			1000								
Categories of co-substrates:											
	Forest		Agriculture		Recycling economy		Food residues		Algae		Non-biomass

Abbreviations: heating rate, HR; retention time, RT; carrier gas, CG; flow rate, FR; biochar yield, BY; nr—not recorded.

A fixed-bed reactor, made from materials like firebricks, steel, or concrete, features a biomass feed system, gas exit, and ash removal unit. In it, materials move downward by gravity, thermally breaking down into biochar, bio-oil, and gases [202]. The advantages of this type of reactor include uniform temperatures, geometry that aids in quantitative analysis, compaction, efficiency in carbon conversion, and the capacity to handle biomass with high ash content, but the drawbacks are its tiny catalytic surface and heating delay [203].

Microwave reactors can heat materials efficiently by dielectric microwave heating. However, this depends on the ability of a particular material to absorb microwave energy and convert it into heat. The electric component of an electromagnetic field causes heating by two main mechanisms: dipolar polarization and ionic conduction [204]. Microwave reactors operate at a specific frequency of 2.45 GHz (corresponding to a wavelength of 12.24 cm). Their limited applicability is a major limitation of microwave reactors, and the use of microwaves as a heating source is restricted to materials that absorb them well [205]. However, with an appropriate material, their advantages include superior heating rates, more uniform interior heating, and reaction completion in a predetermined period [206,207].

For laboratory-scale pyrolysis production, there has been much focus on the use of the tube furnace due to its effectiveness for both pyrolysis/co-pyrolysis processes. The Carbolite Gero tube furnaces, in particular, provide excellent temperature uniformity and are ideal for heating small samples. They come in several designs: the Universal Tube Furnaces, the Split Tube Furnaces, the Rotary Tube Furnaces, and the Gradient Tube Furnaces [208]. Some are designed with an internal work tube, while others have a separate work tube. They use a proportional integral derivative (PID) algorithm to adjust the heating power and control the temperature of the product. They have 10 individual program slots with 24 configuration segments, program scheduling with a real-time clock, data logging to a USB stick, and built-in over-temperature protection.

In assessing the impact of different furnaces on MSS biochar yield, it has been observed that tube furnaces and fixed-bed reactors, which generally exhibit slower heating rates, tend to produce higher yields of biochar. In contrast, rotary furnaces and microwave reactors, which operate at higher heating rates, tend to favor gas production over biochar [209].

According to the data presented in Table 4, the tube furnace is the most frequently used apparatus for both pyrolysis and co-pyrolysis of MSS at the laboratory scale. This preference is likely due to the tube furnace’s superior temperature control and consistent heating along the tube’s length, which are essential for the highly temperature-sensitive pyrolysis reactions [210]. Following the tube furnace in usage popularity are the muffle furnace and the fixed-bed reactor.

The pyrolysis of MSS is also performed at full scale. At this scale, several reactors have been employed for MSS pyrolysis, including the Pyreg pyrolysis reactor, which is a twin-screw carbonization reactor that operates at temperatures between 500 and 800 °C. The pyrolysis gas produced is directly combusted to heat the process, and the exhaust gas is then used in a heat exchanger. This allows the Pyreg process to generally operate in a net-energy zero mode when pyrolyzing MSS. Feedstock for pyrolysis is pre-treated to ensure a maximum particle size of 30 mm to prevent jamming in the screw mechanism [211,212]. A Pyreg reactor is capable of eliminating over 99% of fine particulates in the exhaust gas, meeting strict EU emissions standards [213], and virtually eliminating organic and mineral-based pollutants [214]. The biochar produced by the Pyreg (Dörth, Germany) pyrolysis process is certified to the EBC standard [215], ensuring high quality and climate-beneficial properties for use as a soil amendment, feed additive, or in other applications. These reactors are currently being operated in Austria and Germany. One type of Pyreg reactor that can produce up to 600 tons of biochar yearly, which translates to 1.6 tons daily, and 600 kW of thermal electricity is the PX1500 model (PYREG GmbH, Dörth, Germany) [216]. Another is the Pyreg GmbH P500 (PYREG GmbH, Dörth, Germany), which has an annual capacity of 300 tons of biochar, or 0.8 tons per day [217].

Another reactor used for full-scale pyrolysis is the VOW Hybrid Rotary Kiln Pyrolysis Reactor (VOW ASA, Oslo, Norway). This system combines a rotary kiln pyrolysis reactor with other components like a fluidized bed and pyrolyzes biomass feedstock at temperatures between 500 and 800 °C. Its hybrid design allows the reactor to utilize the pyrolysis off-gases to provide the thermal energy required for the pyrolysis process, thereby improving overall energy efficiency [218]. Operating at a volume capacity of 5 tons/h, it can accommodate large volumes of biomass waste, including MSS. Additionally, the reactor has the capacity to offset up to 30,000 tons/year of CO_2_ emissions [219].

An additional example is the AquaGreen system reactor, which combines a superheated steam dryer and a pyrolysis reactor [220,221,222]. Its integrated design allows the system to utilize the pyrolysis off-gases to fuel the drying process, thereby improving overall energy efficiency [218,221,223]. For MSS pyrolysis, the wet biomass is first dried using a compact and superheated steam dryer, which operates at 200 °C in an oxygen-free atmosphere for 2 h. The dried biomass is then fed to the pyrolysis reactor, which operates at 650 °C in an oxygen-free environment. The pyrolysis process takes 20 min and eliminates harmful pollutants like microplastics, medical residues, PFASs, and PAHs [220].

#### 3.3.2. Mixing Ratio

The mixing ratio, along with other pyrolytic conditions such as heating rate, temperature, and contact time, plays a crucial role in achieving synergistic effects in a co-pyrolysis process [224]. Several ratios have been employed for co-pyrolysis, including 1:1, 1:3, 3:1, 7:3, and 6:4, depending on the purpose of the study. Often, a mixing ratio of 1:1 is used [73,89,96,101,104,122,142]. For instance, a remarkable reduction in leachable HM concentration was observed when 50 wt.% sawdust from furniture was mixed with MSS at 600 °C [86]. Other mixing ratios have also been employed in several studies [70,86,97,124,142]. For instance, a mixing ratio of 3:1 (MSS/walnut shell) exhibited a high capacity for the adsorption of ammonium in neutral or alkaline water [101]. Additionally, gas and bio-oil yields were higher than biochar yield when wheat straw was co-pyrolyzed with MSS at an 80:20 wt.% ratio [105].

The mixing ratio has a substantial effect on biochar yield during MSS co-pyrolysis. Several studies show that increasing the proportion of the biomass co-substrate leads to a decrease in the overall biochar yield compared to MSS alone [17,225]. This is because the co-substrate has a lower solid residue yield than MSS. For instance, Yang et al. found that increasing the ratio of sawdust mixed with MSS from 10% to 50 wt.% led to a decrease in the biochar yield from 73.5% to 63.7% during co-pyrolysis at 300 °C, from 64% to 47.4% at 400 °C, and from 60.1% to 41.9% at 600 °C [86]. Similarly, Jin et al. reported that the co-pyrolysis biochar yield of bamboo sawdust blended with MSS was lower than that of MSS alone [73]. At 400 °C, the biochar yield decreased from 60.6% for MSS alone to 44.5% with a 50 wt.% mixing ratio. At 500 °C, it decreased from 57% to 44.5%, and at 600 °C, from 53.1% to 41.1%. This decline is generally attributed to the higher VM and lower FC content in the co-substrates than in MSS.

#### 3.3.3. Temperature

The temperature is of paramount significance in determining the outcomes of the pyrolysis/co-pyrolysis process. Optimizing pyrolysis temperature within a suitable range is crucial for achieving effective HM stabilization while minimizing environmental risks associated with HM contamination [17]. The choice of temperature is contingent upon the kind of biomass employed, with low temperatures between 400 and 500 °C being the most common. At lower temperatures, secondary pyrolysis reactions are eliminated, while higher temperatures (>500 °C) pave the way for secondary reactions to take place, i.e., further conversion of the primary pyrolysis product through thermal cracking. During secondary reactions, biochar produced from primary reactions undergoes further decomposition into liquid and non-condensable gases, which lowers the biochar yield. Heat is necessary to provide the required temperature to fragment the various bonds in the biomass. Pyrolysis temperature influences the structural changes of MSS biochar. At 300 °C, an aromatic structure gradually emerges. This is accompanied by an enrichment of nitrogen (mainly amino) and oxygen groups, primarily in the form of C=O bonds, within the aromatic structure as the temperature increases. At approximately 500 °C, a graphitized structure begins to appear, coinciding with the disappearance of C=O groups, and the graphitized structure stabilizes at around 700 °C. The aromatic rings present in the biochar provide π-electrons that can form strong bonds with HM ions, improving their stability within the biochar matrix. This interaction helps in the fixation of HMs, preventing their leaching from the biochar [226].

#### 3.3.4. Residence Time

The residence time, i.e., time of pyrolysis of co-pyrolyzed substrates, has a significant effect on biochar quality, particularly in relation to HM immobilization and environmental biochar impact. The polymerization of biomass constituents during biochar production is influenced by residence time. The typical residence time for MSS co-pyrolysis ranges from 20 to 180 min, depending on the reactor. For instance, muffle furnaces have 120 min as the most common residence time for MSS co-pyrolysis [108,114,117,154]. In contrast, tube furnaces typically range from 30 to 180 min, with 60 min and 120 min being the most used [86,123,124,134,227]. Low temperatures and long residence time favor the production of biochar, high temperatures and long residence times favor the production of syngas (higher heating value and lower heating value fuel gas), and moderate temperatures and short residence times favor liquid (bio-oil or tar) production [68].

Increasing the residence time provides a sufficient duration for the constituents to react. In contrast, shorter residence times hinder the repolymerization of constituents, leading to reduced biochar yield. The impact of residence time is not limited to the yield but also extends to the quality and characteristics of biochar, facilitating the development of micro and macro-pores. Studies have reported that longer residence times enhance the pore size in the biochar; however, excessive residence time causes the collapse of the pore structure and decreases the surface area [74]. The effect of residence time is often influenced by variables such as temperature, heating rate, and feedstock composition, complicating the provision of a straightforward concept regarding its role in biochar production [228]. For example, the yield of biochar from co-pyrolysis of MSS and cotton stalks at 600 °C tended to be reduced over residence time: ca. 54% at 30 min, ca. 52% at 60 min, and ca. 48% at 90 min. The co-pyrolysis at residence time of 120 and 150 min gave comparable biochar yield, ca. 46% and 45%, respectively [74].

#### 3.3.5. Heating Rate

The heating rate during biomass pyrolysis and co-pyrolysis is a key determinant of the nature and composition of the final product. The typical heating rate ranges from 10 to 20 °C/min for pyrolysis of MSS alone and, on average, ranges from 5 to 40 °C/min for MSS co-pyrolysis. In some instances, for example, the co-pyrolysis of MSS and willow woodchips involved a heating rate in a wide range of 10, 50, and 100 °C/min. Notably, a study by Al-Mrayat et al. found that the yield of MSS biochar remained consistent even when the heating rate was increased from 5 to 35 °C/min [229]. This indicates that the biochar yield is not significantly impacted by an increase in heating rate within that range.

It is worth noting that the reactor design and configuration for pyrolysis/co-pyrolysis plays a role in the heating rate, as demonstrated in Table 4. For instance, microwave reactors can achieve extremely rapid heating rates, which favors gas production over biochar [230]. Additionally, a semi-batch pyrolysis reactor where the volatiles are removed from the heated zone can lead to thermal degradation occurring primarily in the liquid phase rather than the gas phase [209].

#### 3.3.6. Type of Inert Gas and Carrier Gas Flowrate

Carrier gas used in pyrolysis is mainly used to create an inert environment. However, the flow rate of the carrier gas affects the distribution of the product. When the carrier gas flows through the reactor, it removes the thermal cracking vapors generated by the biomass. It is important to note that the carrier gas is inert and does not participate in any reactions inside the reactor. The reduction in vapor residence time caused by the flowing carrier gas causes a decrease in the biochar yield. However, the residence time is not significantly affected by further increasing the flow rate beyond an optimum value [231]. Nitrogen and argon are commonly used as carrier gases.

Recently, CO_2_ has also been used as an inert gas [75]. Cai et al. found that using CO_2_ during MSS pyrolysis decreases the toxicity of the resulting MSS biochar as compared to using N_2_ [232]. This was attributed to the ability of CO_2_ to promote the stabilization of HMs in biochar. Similarly, when MSS and biomass waste were co-pyrolyzed, Gbouri et al. found that using CO_2_ as a carrier gas instead of N_2_ improves the stability and lowers the leachability of HMs in the resultant biochar [154]. Nevertheless, N_2_ is the most preferred because it is cheaper than other gases and readily available for purging the vapors produced during pyrolysis.

The typical gas flow rate for MSS pyrolysis and co-pyrolysis ranges from 0.01 to 2.0 L/min and 0.05 to 0.5 L/min, respectively. Previous studies, including [229,233,234], have examined the correlation between carrier gas flow rate and biochar yield, revealing that an increase in the former leads to a reduction in the latter [229,235]. The effect, however, is not significant, and a decrease in flow rate results only in a marginal increase in biochar yield. The mechanism behind this relationship has been attributed to the shorter residence time of vapors in the reaction zone when the gas flow rate increases. This shorter residence time hinders the initiation of the polymerization process by the volatile constituents of biomass, driving them out rapidly and consequently reducing the biochar yield. For instance, Feng et al. indicate that increasing the gas flow rate under N_2_ atmospheric pressure during MSS pyrolysis led to a lower yield of the biochar and, in contrast, a higher yield of bio-oil and gas [236]. Similarly, Altıkat et al. also indicated that increasing the gas flow rate during MSS pyrolysis led to a lower yield of the biochar from 34.07% at 0.1 L/min to 32.72% at 0.5 L/min [237].

The choice of carrier gas, particularly CO_2_, can influence the properties of biochars and their suitability for specific applications. For example, the biochars produced from MSS and willow in a CO_2_ environment demonstrated characteristics suitable for agricultural applications and the treatment of contaminated soil and water [17,238].

## 4. Conclusions and Future Perspectives

This review examines the characteristics of co-substrates for MSS co-pyrolysis biochar. It provides a summary of the factors affecting the yield and quality of biochar from MSS pyrolysis and co-pyrolysis, including temperature, heating rate, residence time, and mixing ratio (relevant only for co-pyrolysis). Furthermore, it discusses the types of furnaces employed for MSS pyrolysis and co-pyrolysis processes at both laboratory and large-scale levels, along with their respective advantages and disadvantages. The selection of the co-substrate material plays a crucial role in determining the quality of the co-pyrolyzed biochar. Hence, it is imperative to carefully consider the co-substrate and optimize the co-pyrolysis process when producing biochar for soil application. Co-substrate materials with high carbon content contribute to high SSA and an optimized pore structure. A high content of organic compounds of cellulose, hemicellulose, and lignin in a co-substrate is particularly advantageous for MSS co-pyrolysis. These co-substrates are pivotal in upgrading the properties of MSS-derived biochar, making it suitable for applications like soil amendment and pollutant adsorption.

Future research should expand the exploration of co-substrates for MSS co-pyrolysis, including unconventional substrates, to evaluate their effectiveness in improving biochar properties. Determining the optimal combinations of MSS and various co-substrates could lead to biochar of superior quality and functionality.

It is imperative to thoroughly analyze the micropollutants in MSS, taking into account factors such as the size of WWTP and the type of wastewater treatment technologies used. Such an analysis should also extend to the resulting biochar and focus on how pyrolysis can affect the concentration and leachability of these pollutants and what type of MSS can be selected for co-pyrolysis.

The potential benefits of adding inert gas to pyrolysis processes remain unclear due to a lack of solid empirical evidence. Further research is needed to verify the feasibility of this approach and to fully understand the effects of different gases, their mixtures, and the effects of different atmospheres in combination with co-substrates during MSS co-pyrolysis.

Efforts should also be directed toward the development of MSS pre-treatment methods aimed at reducing the pollutant content prior to co-pyrolysis. Such advances could further improve the quality and safety of the resulting biochar. If these perspectives are taken into account, research and development in the field of MSS co-pyrolysis can lead to more sustainable and environmentally friendly practices and thus make an important contribution to the goals of a circular economy.

A comprehensive investigation of the environmental impact of co-pyrolysis biochar in the context of MSS through a thorough life cycle assessment (LCA) shows considerable potential for future research efforts.

## Figures and Tables

**Table 1:** Frequency of application of MSS treatment in selected EU and non-EU countries and in the USA [13,14].

Treatment		Czechia	Denmark	France	Germany	Greece	Ireland	Italy	Poland	Portugal	Spain	Sweden	U.K.	USA
*Stabilization*														
Aerobic														
Anaerobic														
Lime														
Composting														
*Dewatering*														
Filter press														
Centrifuges														
Belt filter														
*Others*														
Thermal drying														
Solar drying														
Long-term storage														
	Most commonly used		Commonly used							Not used				

**Table 2:** Examples of different feedstocks for biochar production and biochar classification according to EBC requirement [60].

Origin of Feedstock		Examples of Feedstock			Biochar’s Class					
					F	AO	A	U	CM	BM
Agriculture		Energy crops, short rotation woody crops, harvest residues								
		Old straw and grain dust, vegetables, seeds								
Forestry and wood processing		Bark, wood chips, and residues from mechanical processing, sawdust								
Landscape management		Biomasses from nature conservation, landscaping residues								
		Foliage, root stocks								
Recycling residues		Untreated waste wood, paper fiber sludge, residues from industrial biomass								
		Waste paper, waste wood without PVC, heavy metals, or wood preservative								
		Waste wood with PVC and/or HMs, with/without wood preservatives								
Food processing residues on vegetable basis		Pomace, kernels, husk, grist, residues from potatoes, corn, etc.								
		Different residues from food production								
Kitchen waste		Kitchen, canteen, and restaurant residue								
Water maintenance biomass		Aquatic plants and algae								
		Screening, floating debris, mowed material								
Textiles		Cellulose, cotton, and plant fibers, fibers of hemp, sisal								
Anaerobic digestion		Plant-based digestate								
		Digestate from secondary plant biomass								
		Manure digestate, animal byproduct digestate								
Animal byproduct		Bones, manures								
		Other animal byproducts								
Sludges from wastewater treatment		Sludge from municipal wastewater treatment								
		Sludge from other wastewater treatment								
	Permissible		Allowed with some restrictions		Not recommended					

Biochar’s class: **F** Feed, **AO** AgroOrganic, **A** Agro, **U** Urban, **CM** Consumer Materials, **BM** Basic Materials.

**Table 3:** The general effects of feedstock properties on biochar.

Feedstock Property	Main Effects on Biochar
Moisture	Low content is crucial for higher biochar yield
Volatile matter (VM)	High VM content may lead to higher gas and tar yields during pyrolysis, resulting in lower biochar yield
Ash	Low ash content minimizes impurities in the biochar, which can affect its properties such as porosity, surface area, and reactivity. High ash content reduces biochar yield
Fixed carbon (FC)	High FC content contributes to the carbon stability and heating value of the biochar, resulting in a higher biochar yield and quality
Elemental composition (C, H, N, O)	Higher C content and lower O content generally result in higher biochar yield with higher stability, porosity, and surface area
Lignin, cellulose, and hemicellulose	Higher lignin content is associated with higher biochar yield and stability, while cellulose and hemicellulose content can influence biochar porosity and surface area
Particle size and density	Smaller particle size and higher density generally result in higher biochar yield and better control over biochar properties

## Data Availability

Not applicable.
